# Episodic-like memory of rats as retrospective retrieval of incidentally encoded locations and involvement of the retrosplenial cortex

**DOI:** 10.1038/s41598-021-81943-9

**Published:** 2021-01-26

**Authors:** Nobuya Sato

**Affiliations:** Department of Psychological Sciences, Kwansei Gakuin University, 1-1-155, Uegahara, Nishinomiya, Hyogo 662-8501 Japan

**Keywords:** Psychology, Learning and memory

## Abstract

To examine episodic memory in rats, we trained rats to perform two tasks and tested them for memory of past self-behavior without making them expect to be asked about the memory later when encoding. One of the trained tasks was a delayed matching-to-position task in which the rats were required to remember the location of a presented lever. The other was a tone discrimination task in which the rats were required to discriminate between two pure tones. After learning both tasks, the rats were unexpectedly asked the location of the pressed lever after responding to the cue tone in probe trials during test sessions. The rats demonstrated a response bias that suggests that they have the ability to retrospectively recollect their self-behavior, i.e., episodic memory. We next made excitotoxic lesions in the retrosplenial cortex (RSC) and investigated the effects of the lesions on the unexpected recollection. In the rats with lesions of the RSC, the response bias disappeared. This suggests that the RSC has a role in retrospectively answering unexpected questions about self-behavior.

## Introduction

We consciously recollect subjective past experience and re-experience it. Such a mental process is called “episodic memory.” Episodic memory had been considered to be unique to humans. However, after a seminal study in scrub-jays by Clayton and Dickinson (1998)^1^, studies of episodic memory in non-human animals have been developing^2–4^. In animal studies, it is often called “episodic-like memory” since the properties of the memory have been under discussion.

Episodic-like memory in rats has also been reported by several studies. Babb and Crystal (2006)^4^ showed that rats could recognize a combination of the type of food presented (what), the position of an arm in a radial arm maze (where), and the passage of time (when), suggesting the what-where-when aspect of episodic-like memory. Using preference for novelty, several studies showed episodic-like memory in rodents^3,5,6^.

In addition to the aspect of mnemonic contents regarding what, where, and when an event occurred, it has been emphasized that episodic memory is a process of conscious recollection^7^. You can retrospectively recall a memory of an event even if you did not perceive that you would recall the memory of the event afterwards when it occurred. In other words, episodic memory is not intentionally encoded in most cases. To provide evidence for this aspect of episodic memory, an ability to recollect an event that had not been explicitly encoded needs to be demonstrated^7,8^. Thus, episodic memory has been investigated as an ability of incidental memory in animals as well as humans^9–12^. We must not have a subject recognize that it will be afterwards required to recall an event when encoding. In an experimental situation, because recollection needs to occur under conditions in which encoding is incidental, the memory must be tested as an unexpected question. The first animal study about this property of episodic memory was in pigeons^10^. In this study, pigeons retrospectively recollected whether they pecked or not in a situation in which they did not expect to be asked about it when encoding. In a subsequent study, it was reported that pigeons can recollect the location of self-response^11^.

In rats, Zhou et al. (2012)^13^ first showed the ability to recollect information encoded incidentally. Using a radial maze, they trained rats to perform two tasks. One was a five-arm radial maze task. In this task, rats were required to collect the pellets in the arms. The other was a T-maze task. Using three T-shaped arms in the radial maze, the rats were required to make a left turn if they were presented food at the first arm, and to make a right turn if no food was presented at the first arm. In the test trials, the rats visited arms and obtained food (or no food). After that, the rats were required to choose one of two arms that were in the T-maze task. It was expected that they would choose the arm based on whether they had obtained food or not in the first phase of the test trial. In the study, whether food was presented or not was the cue for the response in the second phase of the test trial. In general, however, it takes some time to consume food. It is possible that the aftereffects of the food consumption may serve as the cue for the choice in the T-maze. Accordingly, in the present study, we examined the ability of rats to recollect the location of self-behavior using an approach that was similar to that used by Singer and Zentall (2007)^11^. In an operant chamber set-up, we trained rats to perform two tasks: a delayed matching-to-position task and a tone discrimination task. After that, we tested the ability of the rats to recollect the location of a response lever with a form of unexpected question.

It has been suggested by several studies on humans^14,15^ and rats^13,16^ that the hippocampus is involved in episodic memory. In addition, there are also studies on humans that suggest the involvement of the retrosplenial cortex (RSC) in episodic memory^17–19^. The RSC in primates is a cortical area located in the posterior cingulate cortex around the splenium that is the most posterior region of the corpus callosum. In rodents, the RSC covers most of the posterior medial parietal regions^20^. Studies in humans and rodents suggest that the RSC is involved in spatial cognition^18,20,21^. Although many studies in humans also suggests the involvement of the RSC in episodic memory, there are few studies on this in rodents. Hayashi et al. (2020)^6^ reported the effects of RSC lesions on episodic-like memory in rats using an object-exploring procedure. In this study, we examined the effect of the lesions of the RSC on incidental recollection to manifest the involvement of the RSC in episodic-like memory.

## Results

### Behavioral training

**Figure 1:**
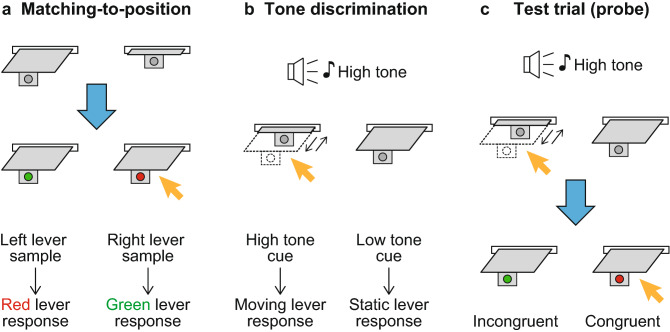
Task procedures. (**a**) An example of a trial of the delayed matching-to-position (DMP) task. In this trial, the left lever was presented as a sample. After the rat pressed the sample lever followed by the delay, both levers with tips that had LED lights were presented. In this case, the rats were required to press the right red lever (the orange arrow). (**b**) An example trial of the tone discrimination task. The rats were required to discriminate between 2 kHz (low) and 4 kHz (high) pure tones. One of the two levers was repeatedly moved in and out with a period of 2 s, and the other was static. In this case, the high tone was presented as a cue, and the correct response was pressing the left moving lever (the orange arrow). (**c**) An example of a probe trial in the test session. In this case, the high tone was first presented and the moving and static levers were presented at the left and right sides, respectively. The rats were required to press the corresponding lever according to the rules of the tone discrimination task (the upper orange arrow). Pressing one of the levers made both levers retract. After the 2-s delay period, both levers that had tips illuminated with the red or green LED were presented for 10 s. According to the rules of the DMP task in (**a**), the congruent response is that to the red right lever (the lower orange arrow), and the response to the green left lever is incongruent.

To examine episodic-like memory in rats, we trained the rats to perform two tasks: a delayed matching-to-position (DMP) task and a tone discrimination task (Fig. 1). In each trial of the DMP task, one of two levers (left/right) was first presented as a sample. If the rat pressed the sample lever, it was retracted. After a 5-s delay, the left and right levers were presented. The color of a light-emitting diode (LED) attached to the tip of each lever was a test stimulus. The rats were required to press the lever for which the color of the LED corresponded to the position of the sample lever (Fig. 1a, Table 1).

**Table 1:** Combinations of the cue and test stimulus in the tone discrimination and delayed matching-to-position tasks.

Combination no.	Cue of the tone discrimination		Sample lever of the delayed matching-to-position	
	High	Low	Left	Right
1	Moving	Static	Green	Red
2	Moving	Static	Red	Green
3	Static	Moving	Green	Red
4	Static	Moving	Red	Green

In the tone discrimination task, the rats were required to discriminate two tones. In each trial, one of the tones was presented. One of two levers in the experimental box was repeatedly moved in and out with a period of 2 s, and the other lever was static. The rats were required to press the lever corresponding to the cue tone (Fig. 1b, Table 1).

In the first phase of the task learning, the majority of the rats engaged in the DMP task, but some engaged in the tone discrimination task. The rats needed 39.8 ± 18.0 (mean ± SD) daily sessions to reach the learning criterion, 80% correct responses in a session that consisted of 160 trials. All of the rats reached the criterion in the first phase. In the second phase, they engaged in both tasks, and all except for three reached the criterion in 101.4 ± 86.4 sessions (Supplementary Fig. S1).

### Test for retrospective recollection of a location of a self-response

After the training, test sessions were carried out. In the test sessions, both tasks were alternatively presented in approximately 20-trial blocks. During the blocks of the tone discrimination task, we inserted probe trials (Fig. 1c). A probe trial consisted of two parts. The first part used the same procedure as the tone discrimination task, except that no reward was granted. Briefly after the rat’s response, the second DMP part started, with both levers presented. The tips of the levers were illuminated with red or green LEDs. All combinations of the tone (high/low), the side of the moving (or static) lever (left/right), and the side of the red (or green) LED (left/right) were presented in a test session, i.e., there were eight probe trials in a test session (Table 2). The rats had never experienced the presentation of the levers with the illuminated LEDs after pressing the lever in the tone discrimination trials. The rats never expected to be asked to which lever they had responded after the tone discrimination procedure, and thus were required to recall it retrospectively.

**Table 2:** Combinations of cue tone, lever dynamic status, and lever LED status in probe trials, and assumed response for each trained combination.

Presented tone	Presented lever in the tone discrimination		Presented lever in the delayed matching-to-position		Assumed response			
	Left	Right	Left	Right	1	2	3	4
High	Moving	Static	Green	Red	Left	Right	Right	Left
			Red	Green	Right	Left	Left	Right
	Static	Moving	Green	Red	Right	Left	Left	Right
			Red	Green	Left	Right	Right	Left
Low	Moving	Static	Green	Red	Right	Left	Left	Right
			Red	Green	Left	Right	Right	Left
	Static	Moving	Green	Red	Left	Right	Right	Left
			Red	Green	Right	Left	Left	Right

The numbers in the columns in the “assumed response” are the combination number shown in Table 1.

**Figure 2:**
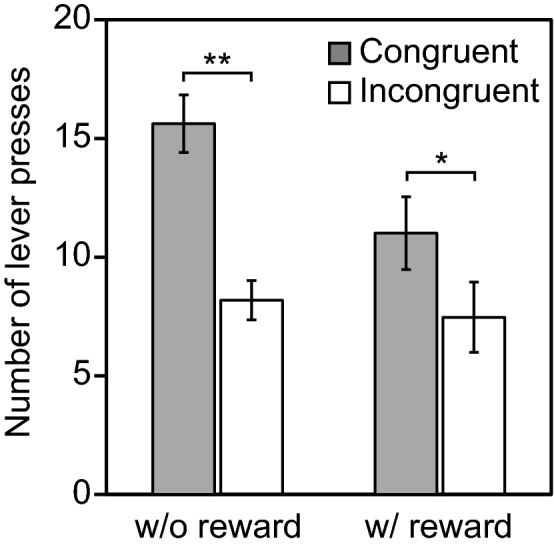
The results of the tests. The gray and white bars indicate the number of responses to the congruent and incongruent levers, respectively. The left is the result of the original test in which a reward was not granted after the correct response in the tone discrimination part in the probe trials. The right is the result of the additional test in which a reward was granted after the correct response in the tone discrimination part. The error bars indicate the standard error of means. Asterisks indicate significant difference, *p < 0.01; **p < 0.0001.

In the test trials, the numbers of responses to each lever were counted. If the rats can recall their last self-response, it is predicted that they will respond correctly in the DMP procedure, i.e., regarding the lever responded to in the previous tone discrimination part as a sample for the DMP part, and by responding to the lever for which the color of the LED matches the DMP rule. To examine the prediction, we compared the number of lever pressings for the congruent and incongruent levers (Fig. 2, Supplementary Fig. S2). The results were in agreement with the prediction. The number of responses to the congruent lever was significantly larger than that to the incongruent lever (*t*(20) = 5.75, *p* < 0.0001). The proportion of times the congruent lever was pressed (0.65 ± 0.02, mean ± SEM) was significantly higher than the chance level (50%, *t*(20) = 6.85, *p* < 0.0001). This suggests that the rats retrieved their self-behavior even when it was asked unexpectedly. It was not explicitly to-be-encoded information when the rat responded in the tone discrimination part of the probe trials. The rats should have retrospectively recollected it, i.e., episodic recollection, when the two levers with the LED lights were presented in the matching-to-position part of the probe trials.

### Exclusion of a simple association explanation

The rats could have used another strategy. One explanation for the results could be that the color of the LED was simply associated with approach or avoidance responses instead of indicating recollection of past self-behavior. To exclude this possibility, we carried out another test in some rats. In this test, the procedure was almost the same as in the test sessions aforementioned except for presenting a food pellet (or only a clicker noise that had been presented with the food pellet) after the tone discrimination part in the probe trials. In this procedure, the rats left the lever and approached the food cup. This should cancel out a strategy in which they simply approached or avoided the lever according to the color of the LED. In spite of the procedure that inhibited the simple association strategy, the rats demonstrated response tendencies similar to those in the original test (Fig. 2). The number of responses to the congruent lever was significantly larger than that to the incongruent lever (*t*(7) = 3.53, *p* < 0.01). The proportion of times the congruent lever was pressed (0.62 ± 0.03, mean ± SEM) was significantly higher than the chance level (*t*(7) = 3.46, *p* < 0.05). This suggests that the simple association explanation can be excluded.

### The effect of lesions of the retrosplenial cortex

**Figure 3:**
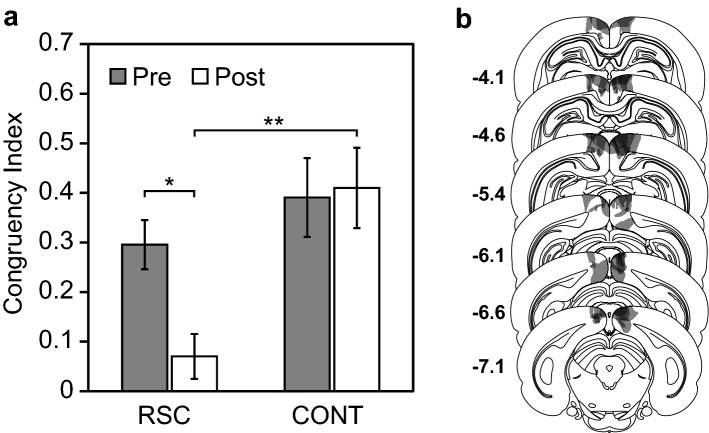
The effect of the RSC lesion. (**a**) Pre- (gray bars) and post-operative performance (white bars) of the rats with the RSC lesions (left) and of the control rats (right) in the probe trials of the test sessions. The error bars indicate the standard error of means. Asterisks indicate significant simple effects, *p < 0.005; **p < 0.001. (**b**) Histological results of the RSC lesions. Coronal sections illustrating the extent of the lesions of the RSC for each rat (shaded gray areas). The numbers indicate the distance (in mm) posterior to the bregma^52^. Bilateral damage to the RSC was found in the rats of the RSC lesion group. In most cases, the damage was limited to the RSC area. However, in some cases, the damage extended into the neighboring area.

We also carried out the test in the rats with the damaged retrosplenial cortex (Fig. 3). After the surgery, the rats with the RSC lesions did not demonstrate the response biases in favor of the congruent lever in the probe trials, whereas the control rats still demonstrated the biases (Fig. 3a). In order to compare the performances of the lesioned and control rats, we calculated a congruency index that is the difference in the number of responses between the congruent and incongruent levers divided by their sum. The comparison between the pre- and post-operative congruency indices was statistically significant in the rats with the RSC lesions but not in the control rats (Fig. 3a). A two-way mixed ANOVA for the group as a between-subjects factor and for the test period as a within-subjects factor revealed significant main effects of group (*F*(1,12) = 8.38, *p* < 0.05) and test period (*F*(1,12) = 5.01, *p* < 0.05). The interaction was also significant (*F*(1,12) = 7.06, *p* < 0.05). A post-hoc analysis revealed significant simple effects of group in the post-operative period (*F*(1,24) = 14.87, *p* < 0.001) and of test period in the RSC group (*F*(1,12) = 11.98, *p* < 0.005). This suggests that the RSC has a role in recalling self-behavior in situations where it was not specified that it would be asked later when encoding.

## Discussion

The present study examined the ability of rats to retrospectively recollect information about location. The results suggests that rats can retrieve the location of past self-behavior in situations in which they could not have expected to be asked about it when encoding. In the probe trials, the rats responded more to the lever that corresponded to the correct response of the matching-to-sample rule. The rats had never experienced the sequence such that the tone discrimination procedure was followed by the matching-to-position procedure in a trial. Thus, the rats could not have expect to be asked the location of the lever that was pressed in the tone discrimination part and had to retrospectively recollect it in the DMP part of the probe trial. The result of the test suggests that rats can retrospectively recollect their past behavior, i.e., have an episodic-like memory^10,11^. This is consistent with previous studies reporting incidental memory in rats^13,22^. In one of these studies, rats were required to make a choice depending on whether they had obtained food rewards or not in the first part of a test trial^13^, whereas the rats in the present study were required to recollect the location of their response in the first part of the probe trial. Because of this procedure, the present study suggests that rats, similar to pigeons in a previous study^11^, can unexpectedly encode an external cue (location) instead of an internal cue (food consumption) and recall it afterwards, and adds more evidence regarding memory in rodents.

In the additional test in which a reward was presented after pressing the lever in the tone discrimination part of the probe trial, the rats demonstrated a response bias similar to that in the original probe test. This additional procedure made the rats face the food cup and reset their attention to the lever’s location before they were required to respond in the DMP part of the probe trial. This result cannot be explained by a simple association between the color of the LED and approach or avoidance responses, and supports the interpretation that the behavior of the rats in the present study reflects their capacity for episodic-like memory.

The retrospective recollection in the present study was operationally defined as the response to the lever corresponding to an unexpected question about the location of past self-behavior. Under the definition, the representation of the location is highly likely to be activated by the recollection process. It must not be activated by information available at the time point when the subject is required to recollect it. To fulfill it, the rats in the present study leaned two tasks, and in one of the tasks, the tone discrimination task, the location of the lever was never associated with the task demand. In addition, there were two possible locations where the rats retrieved in the DMP part of a probe trial, and they had to adjust their responses depending on the type of the probe trial. It is not the case that the representation of a specific location was activated by some information available at the time of the DMP part of the probe trial. However, the rats were required several times to encode the location of the lever in the blocks of the DMP task within the same test session. This might have implicitly induced activation of the encoding process of the location of the lever in a probe trial even if the rats had no experience of encoding the location of the response lever in the tone discrimination task. If this is the case, it is predicted that the smaller the number of trials between the probe trial and the transition from the DMP block to the tone discrimination block, the better the performance will be; i.e., the rats will press the congruent lever more in the probe trials. To investigate this, the correlation between the congruency indices and the number of trials conducted from the beginning of the tone discrimination block to a probe trial was calculated. There was no significant correlation between them (*r* = − 0.02). This suggests that it is unlikely that the encoding process of the location of the lever continued until the probe trials occurred, but it does not deny the possibility completely. Further studies are needed to prove the existence of episodic memory in animals.

The present results of the test in the rats with the RSC lesions suggest that the RSC is involved in the process of episodic recall. Although previous studies suggest that the RSC has a role in spatial cognition^20^, the rats with the RSC lesions in the present study could solve the DMP task as well as the tone discrimination task independently at the level of more than 80% correct responses. This suggests that the process needed to produce the congruent responses in a probe trial is different from that needed to solve the DMP task itself, and that the intactness of the RSC might not be necessary for such basic perceptual and memory functions. Hayashi et al. (2020) reported that RSC lesions impaired episodic-like memory, especially its temporal aspect. In that study, they examined rats’ mnemonic ability to recall elementary What, Where, and When information independently, as well as the combination of all three aspects, and found effects of lesions of the RSC on the When memory as well as on the integrated episodic-like memory. The present results suggest that the RSC may be involved in retrospectively recollecting a past incidental event. To distinguish a past event from the current situation, some temporal process is needed. The RSC might be involved in such a temporal aspect of episodic memory.

As for a recollection process, the method using the form of a receiver operating characteristic (ROC) has been known. Recognition is thought to be divided into two memory processes (recollection and familiarity), and one can see in the shape of the ROC curve whether an organism makes a familiarity-based judgement (curvilinear ROC function) or a recollection-based judgement (linear ROC function)^23^. Fortin et al. (2004)^24^ found a linear ROC for odor memory in rats, and suggested that the rats judged their odor memory based on recollection instead of familiarity (but see Wixted and Squire 2008)^25^. Application of this to incidental memory may lead to greater understanding of episodic recollection. In addition, through examination of the effects of temporary inactivation using methods like optogenetics, we will be able to manifest whether the RSC has an important role in encoding or in retrieval.

The RSC is also involved in navigation^20,21,26,27^. The RSC contains head direction cells^28,29^ and navigation-related cells^26,27^. Several studies suggest that it plays an important role in route knowledge^21,26,27^. Route knowledge is thought to be a sequential representation of a specific behavior at a specific location^30,31^. In addition, there is a study that reported the involvement of the RSC in object recency memory^32^. These findings may also suggest a relationship between the RSC and temporal order or sequential information processing. However, the details have not yet been manifested and further study is needed.

The RSC has neural connections with the subiculum, which is a major output area of the hippocampus^33–35^, and directly with the hippocampal CA1^34,36,37^. The hippocampus is known as a strong candidate for a neural substrate of episodic memory^14,38,39^. Hippocampal neurons demonstrate place selectivity known as place cells^40,41^ as well as temporal selectivity^42^, suggesting that the hippocampus contains spatio-temporal information. Aggleton and Brown (1999)^39^ highlighted the role of the hippocampal and anterior thalamic nuclei in episodic memory. The RSC together with these two areas is a part of the Papez circuit^43^. The areas contained in the Papez circuit are likely to be needed for episodic memory^44^. There are many unresolved issues, e.g., whether there is a difference in the role in episodic memory between the hippocampus and the RSC, and if so, what kind of difference there is.

Lesion sites of the present study extended into neighboring areas. At the least, the rats could show normal behavior in the DMP and tone discrimination tasks. Their impairment was restricted to the test for the recollection of incidental encoded memory. The subareas of the RSC have different connections with the other areas^44^. A detailed investigation as to the functional differences of the subareas of the RSC will provide deeper understanding of its involvement in episodic memory.

To examine the ability of episodic recall in rats, we used two tasks: the DMP and tone discrimination tasks. To simultaneously satisfy the learning criteria for which the rats demonstrate over 80% correct responses in both tasks, we needed a long period of training, as long as approximately 140 days. Interference between the tasks might have impeded the learning of the tasks^45,46^. Even in healthy humans, execution of multiple tasks is difficult. It might have been too much of a burden for rats. However, it is also suggested that animals can solve dual tasks without a much longer period of training^47^. A study that examined episodic memory in rats using a radial arm maze succeeded in making the rats learn two kind of tasks within a relatively short period^13^.

The present study concerned the rats’ ability of recollection that is an aspect of episodic memory^48,49^. In addition to this, episodic memory has originally been thought to have another aspect, what-where-when memory^48–50^. The function of recollection highlights the retrieval process of episodic memory while what-where-when memory highlights the property of encoded information. Understanding of episodic memory needs to clarify both functions. For that, examining the RSC’s functions may provide a key because the RSC is involved in what-where-when memory^6^ and temporal order^51^ as well as in retrospective recollection of an incidental event, which the present study suggests. Further studies using several approaches will provide better understanding of the functions of the RSC in the future.

## Methods

### Subjects

The subjects were 21 male Long-Evans rats (Japan SLC, Hamamatsu). At the beginning of the experiment, they were 17 weeks old and were weighed 395 g on average. To control their access to food, the rats were individually housed in a stainless wire cage (200 × 250 × 187 mm). All rats were deprived of food to be maintained at 85% of their ad-libitum weights, but were allowed free access to water during all experiments. Twelve of the rats were on a 16/8 h light/dark cycle (lights were on from 8:00 to 24:00) and the rest were on a 12/12 h light/dark cycle (lights were on from 9:00 to 21:00) with controlled temperature (23 °C) and humidity (60%). All experiments in this study were approved by the Animal Experimentation Committee of Kwansei Gakuin University, and were complied with the “Kwansei Gakuin University Regulations for Animal Experimentation,” the “Fundamental Guidelines for Proper Conduct of Animal Experiment and Related Activities in Academic Research Institutions” prescribed by the Ministry of Education, Culture, Sports, Science and Technology of Japan, and the ARRIVE guidelines.

### Apparatus

All experiments were carried out in operant boxes. In each box, there were a food cup and two retractable levers (H23-17R, Coulbourn Instruments) on the front panel. Each lever had a light-emitting diode (LED) on the tip, which could present several colored illuminations. Stimulus sounds were provided by a programmable audio generator (ANL-926, Med Associates). All experimental devices were controlled through interface devices (DIG-716, Med Associates) using an operating software (MED-PC IV, Med Associates).

### Behavioral procedures

We trained rats to perform two tasks: a delayed matching-to-position (DMP) task and a tone discrimination task (Fig. 1). In each trial of the DMP task, one of two levers (left/right) was first presented as a sample. If the rat pressed the sample lever, it was retracted. The lever was presented for a maximum of 60 s. If the rat did not press it in that period, the trial was aborted. After a delay period, the left and right levers were presented. The color of the LED (red/green) attached to the tip of each lever was a test stimulus. The rats were required to press the lever for which the color of the LED corresponded to the position of the sample lever (Table 1). Twelve of the 21 rats were required to respond to the lever with the red LED if the sample was the left lever, and to the lever with the green LED if the sample was right lever. The remaining rats were required to respond with the reverse correspondence. When the rats made the correct response, they were granted a 45-mg food pellet (F0021, Bio-Serv) as a reward together with a 500-ms clicker noise. In the initial period of the training, the required number of times the lever had to be pressed to respond was one, and this was gradually increased to five. The delay period also started with 0 s and was gradually extended to 5 s. If the rats responded to the wrong lever, they were granted a 20-s timeout period. The intertrial interval variably ranged from 5 to 11 s (8 s, on average).

In the tone discrimination task, the rats were required to discriminate between 2 and 4 kHz pure tones. In each trial, one of the tones was presented. Four seconds after the tone onset, the two levers were inserted to the box. One of the levers was repeatedly moved in and out with a period of 2 s (1.7 s inside and 0.3 s outside of the box), and the other lever was static after the insertion. The rats were required to press the lever that corresponded to the cue tone (Table 1). Nine of the 21 rats were required to press the moving lever if the cue was the 2 kHz tone and to press the static lever if the cue was the 4 kHz tone. The remaining rats were required to respond with the reverse correspondence. The procedures for the reward, the timeout, and the intertrial interval were the same as in the DMP task.

First, the rats were trained to perform one of the two tasks with 160 trials in a daily session. After satisfying the learning criterion of 80% correct responses, training on the other task started. In a 160-trial session divided into two 80-trial blocks, the rats performed the same task in one block. In the other block, they were trained to perform the other task. After satisfying the 80% criterion, a session was divided into four blocks: two for the DMP task and two for the tone discrimination task. Again, after satisfying the criterion, the session was divided into eight blocks. The training continued until the criterion was satisfied for this condition.

After the training, the test sessions were carried out. In the test sessions, both tasks were alternately presented with approximately 20-trial blocks. During the blocks of the tone discrimination task, probe trials were inserted. A probe trial was started with the high or low tone. Four seconds after the tone onset, the moving and static levers were presented. The rats were required to press one of the moving and static levers according to the rules of the tone discrimination task. When the rat pressed one of the levers, both levers were retracted. If the rat pressed the wrong lever, the trial was aborted and a 20-s timeout period was granted. After the 2-s delay period, both levers with tips illuminated with a red or green LED were presented for 10 s. During the 10-s period, the response of the rats was measured. All combinations of the tone (high/low), the side of the moving (or static) lever (left/right), and the side of the red (or green) LED (left/right) were presented in a test session, i.e., there were eight probe trials in a test session (Table 2). Between each probe trial, there were 19 trials of the standard task (the DMP or tone discrimination task) on average. Four sequences of the trials were used for the test sessions. In the next few days of the test session, the two-task trainings with 20-trial blocks were carried out until the rats again satisfied the criterion.

### Data analysis

In a probe trial of the test sessions, the number of times each lever was pressed during the 10 s in the DMP part was counted. The values were separately averaged for the congruent and incongruent responses. A congruent response is defined as pressing the lever that corresponds to the correct response when the response lever in the last tone discrimination part is regarded as a sample of the current DMP part. For the analysis, we calculated the congruency index using the following formula.

$$Congruency\; index = \frac{{R_{cong} - R_{incong} }}{{R_{cong} + R_{incong} }}.$$

Here, *R*_*cong*_ is the number of responses to the congruent lever and *R*_*incong*_ is the number of responses to the incongruent lever. This index takes values from -1 to 1, and a larger value indicates that the rats responded more to the congruent lever.

### Surgery

In the surgery for the present study, some rats were anesthetized with sodium pentobarbital (50 mg/kg, i.p.), and some were anesthetized with isoflurane. For the isoflurane anesthesia, the rats were first injected with a ketamine-xylazine mixture (40 mg/kg and 5 mg/kg, i.p., respectively) for the induction. Then, they were anesthetized with 5% isoflurane at a flow rate of 0.5 L/min. After 2–3 min, the concentration of the isoflurane and the flow rate were maintained with 2–3% and 0.3 L/min, respectively. After the anesthesia, each rat was placed in a stereotaxic apparatus (David Kopf Instruments). The excitotoxic lesions of the RSC were produced by *N*-methyl-d-aspartate (NMDA; Sigma-aldrich). The stereotaxic coordinates of the six target sites were as follows: AP − 4.7, ML ± 0.6 (from bregma), DV − 1.0 (from dura); AP − 5.8, ML ± 0.8, DV − 1.2; AP − 6.9, ML ± 0.9, DV − 1.5. The NMDA was dissolved in 0.1 M phosphate buffer saline (pH 7.4) at a concentration of 17.5 mg/mL. Through a glass pipette made by a puller (PC-10, Narishige) and attached to a 5-µL microsyringe, we bilaterally injected 0.3 µL of the NMDA solution into each target site with a flow speed of 0.3 µL/min. After the penetration of the glass pipette, it was left in place for 5 min before the injection, and after the injection, it was left in place again for 3 min. The rats in the control group received the same procedure as the lesioned rats except for the injection of NMDA. After the surgery, the rats were kept in the homecage for at least one week for recovery. Two rats from the control group died during the surgery.

### Histology

At the end of the experiment, all of the lesioned rats were deeply anesthetized with an overdose of sodium pentobarbital (120 mg/kg, i.p.) and transcardially perfused with saline followed by 10% buffered formalin solution. Their brains were removed from their skulls and stored in a 10% formalin solution for one day. Then the brains were submerged in 10% followed by 30% sucrose solutions. They were frozen and sectioned at 40-µm intervals and stained with cresyl violet or hematoxylin/eosin for investigation of the lesion sites.

## Supplementary Information

Supplementary Figures.

## Data Availability

The datasets generated during and/or analysed during the current study are available from the corresponding author on reasonable request.
