# Supplementary material for: Episodic-like memory of rats as retrospective retrieval of incidentally encoded locations and involvement of the retrosplenial cortex
Source: Sci Rep. 2021 Jan 26;11:2217. doi: 10.1038/s41598-021-81943-9 (PMC7838390; doi:10.1038/s41598-021-81943-9)
Supplement: Supplementary file 1 — Supplementary Figures. [file 41598_2021_81943_MOESM1_ESM.pdf]

Supplementary information for

Episodic-like memory of rats as retrospective retrieval of incidentally encoded locations and involvement of the retrosplenial cortex

Nobuya Sato

Department of Psychological Sciences, Kwansei Gakuin University

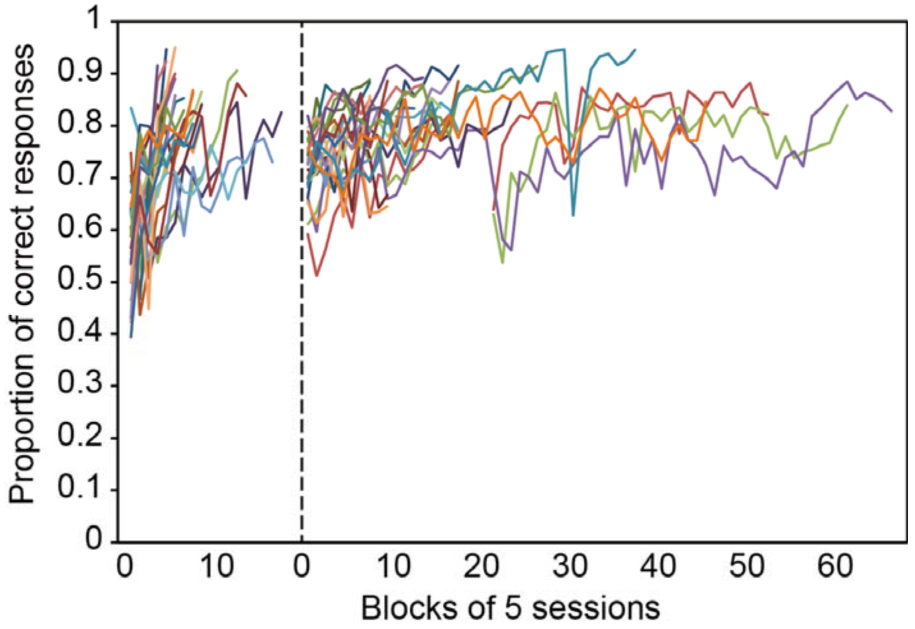

Fig. S1.

The performance of each rat in the learning phases. The left of the vertical dashed line is the first phase of the learning and the right is the second phase.

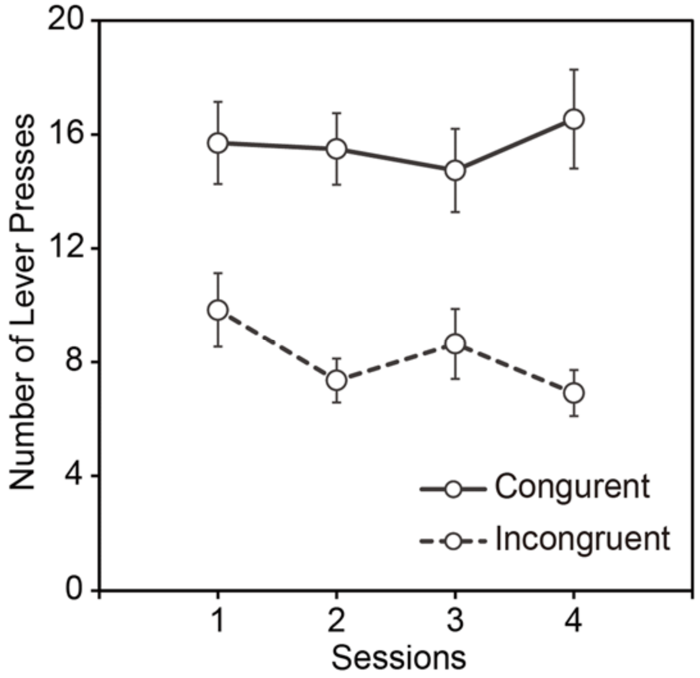

Fig. S2.

The mean number of lever pressing to the congruent and incongruent levers in each probe test session. A two-way ANOVA revealed a significant main effect of congruency ($F(1,20) = 33.04$, $p < 0.0001$). There was neither a main effect of session ($F(3,60) = 1.06$, $p = 0.37$) nor a congruency x session interaction ($F(3,60) = 1.80$, $p = 0.16$).
